# Efficient management of the nitritation-anammox microbiome through intermittent aeration: absence of the NOB guild and expansion and diversity of the NOx reducing guild suggests a highly reticulated nitrogen cycle

**DOI:** 10.1186/s40793-022-00432-2

**Published:** 2022-07-22

**Authors:** Alejandro Palomo, Daniela Azevedo, María Touceda-Suárez, Carlos Domingo-Félez, A. Gizem Mutlu, Arnaud Dechesne, Yulin Wang, Tong Zhang, Barth F. Smets

**Affiliations:** 1 Microbial Ecology and Technology Lab, Department of Environmental Engineering, Technical University of Denmark, Kgs Lyngby, Denmark; 2 Environmental Microbiome Engineering and Biotechnology Laboratory, Department of Civil Engineering, The University of Hong Kong, Hong Kong SAR, People’s Republic of China; 3 Present Address: School of Environmental Science and Engineering, Southern University of Science and Technology, Shenzhen, Guangdong Province China; 4 Present Address: Hydrotech - Veolia Water Technologies, Vellinge, Sweden; 5 Present Address: Department of Environmental Science, University of Arizona, Tucson, AZ USA

**Keywords:** Nitritation, Anammox, Ammonia, Nitrite, Brocadia, Nitrosomonas, Nitric oxide

## Abstract

Obtaining efficient autotrophic ammonia removal (*aka* partial nitritation-anammox, or PNA) requires a balanced microbiome with abundant aerobic and anaerobic ammonia oxidizing bacteria and scarce nitrite oxidizing bacteria. Here, we analyzed the microbiome of an efficient PNA process that was obtained by sequential feeding and periodic aeration. The genomes of the dominant community members were inferred from metagenomes obtained over a 6 month period. Three *Brocadia* spp. genomes and three *Nitrosomonas* spp. genomes dominated the autotrophic community; no NOB genomes were retrieved. Two of the *Brocadia* spp. genomes lacked the genomic potential for nitrite reduction. A diverse set of heterotrophic genomes was retrieved, each with genomic potential for only a fraction of the denitrification pathway. A mutual dependency in amino acid and vitamin synthesis was noted between autotrophic and heterotrophic community members. Our analysis suggests a highly-reticulated nitrogen cycle in the examined PNA microbiome with nitric oxide exchange between the heterotrophs and the anammox guild.

**Supplementary Information:**

The online version contains supplementary material available at 10.1186/s40793-022-00432-2.

## Introduction

Predicting and managing the composition and function of microbial communities is heralded as the holy grail of microbial ecology [1]. Composition can be imposed when working under aseptic conditions, but is challenging when communities are open, the typical scenario for most ecology-relevant and technology-interesting microbial communities. While rational engineering of microbiomes is an active field of research, it is recognized that we still lack the required mechanistic understandings to design microbiomes based on a priori knowledge [2].

On the other hand, heuristic approaches based on manipulation of environmental conditions, have a long history of success in managing microbial communities towards specific functions in food, agriculture, and environmental applications. While those management practices do not rely on first principle understanding of microbial community assembly, unravelling those communities, and seeking links with management constraints might yield insights on which to build new testable theories [2].

The common, and intuitively simple, conditions that one can impose on a microbial community are the provision/fluxes of specific electron donors *versus* electron acceptors and of macro-or micronutrients; provision or limitation of these will impose selective pressures and enrich communities with the desired phenotypes. In addition, community control may be further facilitated by spatio-temporal gradients that naturally occur or are artificially imposed. Gradients are especially valuable when they permit the establishment of multiple redox conditions over short spatial or temporal scales that allow the co-occurrence of functional groups that require mutually exclusive environmental conditions; the characteristic of microbial aggregates and biofilms [3, 4].

Here, we examine the management of a microbial community with the functional property of complete autotrophic conversion of ammonia (NH_3_) to dinitrogen gas (N_2_), also known as the partial nitritation/anammox process (PNA) [5]. The simplest functional PNA community would consist of only two functional groups: one that performs aerobic NH_3_ oxidation (using O_2_ as terminal electron acceptor (TEA) and forming NO_2_^−^, aerobic ammonia oxidizing prokaryotes or AOBs) and one that performs anoxic NH_3_ oxidation (using NO_2_^−^ as TEA and forming N_2_, anaerobic ammonia oxidizing prokaryotes or AnAOBs). The PNA process relies on the provision of controlled (limited) supply of oxygen and the spatial or temporal variation in redox conditions [6, 7]. Yet, the PNA process remains tested by microbial community management, especially the suppression of the aerobic NO_2_^−^ oxidation guild (aerobic nitrite oxidizing prokaryotes or NOBs) [7, 8]. Indeed, all metagenetic and metagenomic analyses of PNA communities to date indicate the persistent presence of NOBs; and their excessive presence would deteriorate or collapse the PNA process [8, 9]. Selection for AOB against NOBs has variously relied on growth inhibition by free ammonia or nitrous acid [10] or oxygen limitation driven by the low O_2_ affinity of the NOB [9, 11–13]. In addition, while autotrophic NH_3_ and NO_2_^−^ oxidizers are the only microbes that can grow on the influent devoid of organic carbon and are essential for a functional PNA process, their metabolism and decay will result in release of organic byproducts. As a result, heterotrophic microbes are inevitable and their abundance and potential symbiosis with autotrophs has previously been found [14, 15], yet their functional contribution is ill-documented.

We document here that periodic and limiting provision of oxygen (via intermittent aeration) to a granule-based reactor can result in complete elimination of NOBs from the community performing the PNA process. We examine the consequences of this operation on the community structure, with specific attention on alternate pathways for NO_x_ (nitrogen oxides) metabolism. We identified a representative set of high-quality MAGs representing most of the metagenome, a few highly abundant AOB and AnAOB, the absence of NOB, and a diversity in NOx respiratory abilities across the heterotrophic MAGs. Genome analysis suggests a highly reticulated network with possibility for NO exchange between autotrophs and heterotrophs and strong evidence of auxotrophies distributed across the community members.

## Results

### Process performance

Prior to this experimental phase, the reactor had been operating for nearly 19 months, and the last five months using a consistent regime that included three aerated and non-aerated intervals (30% and 70% of the react phase respectively): total nitrogen (TN) removal efficiency was around 84.5% [16]. During the reported phase, the frequency of redox switching increased on a monthly basis from 3 to 4, 6, 8, 10, 16 and 25 per cycle. Throughout these six months of operation, process performance deteriorated slightly with removal rates varying from 86 ± 4% TN in early phase and reaching 75 ± 4% at highest switching frequency (increasing oxygen loading recovered 85% efficiency). No residual nitrite was ever detected in the effluent and the ratio of nitrate produced per unit of ammonium removed (R_NatTot_ [17]) remained below 0.13, pointing at AnAOB as the only generators of NO_3_^−^ [18]. Further details are reported elsewhere [16].

Community analysis via *nxr* and 16S rRNA gene targeted qPCR [16] and 16S rRNA gene amplicon sequencing (Additional file 1: Fig. S1) indicated extremely low abundances of taxa containing typical nitrite oxidizing bacteria (predominantly in smallest aggregates (< 90 µm)) *Nitrobacter* spp. and *Nitrospira* spp., but high abundance of taxa comprising AnAOB (*Brocadia* spp.) and AOB (*Nitrosomonas* spp.). AOB fractions increased with redox switching frequency, but only in the largest aggregates (> 600 µm) [16].

### Quality of MAGs

**Fig. 1:**
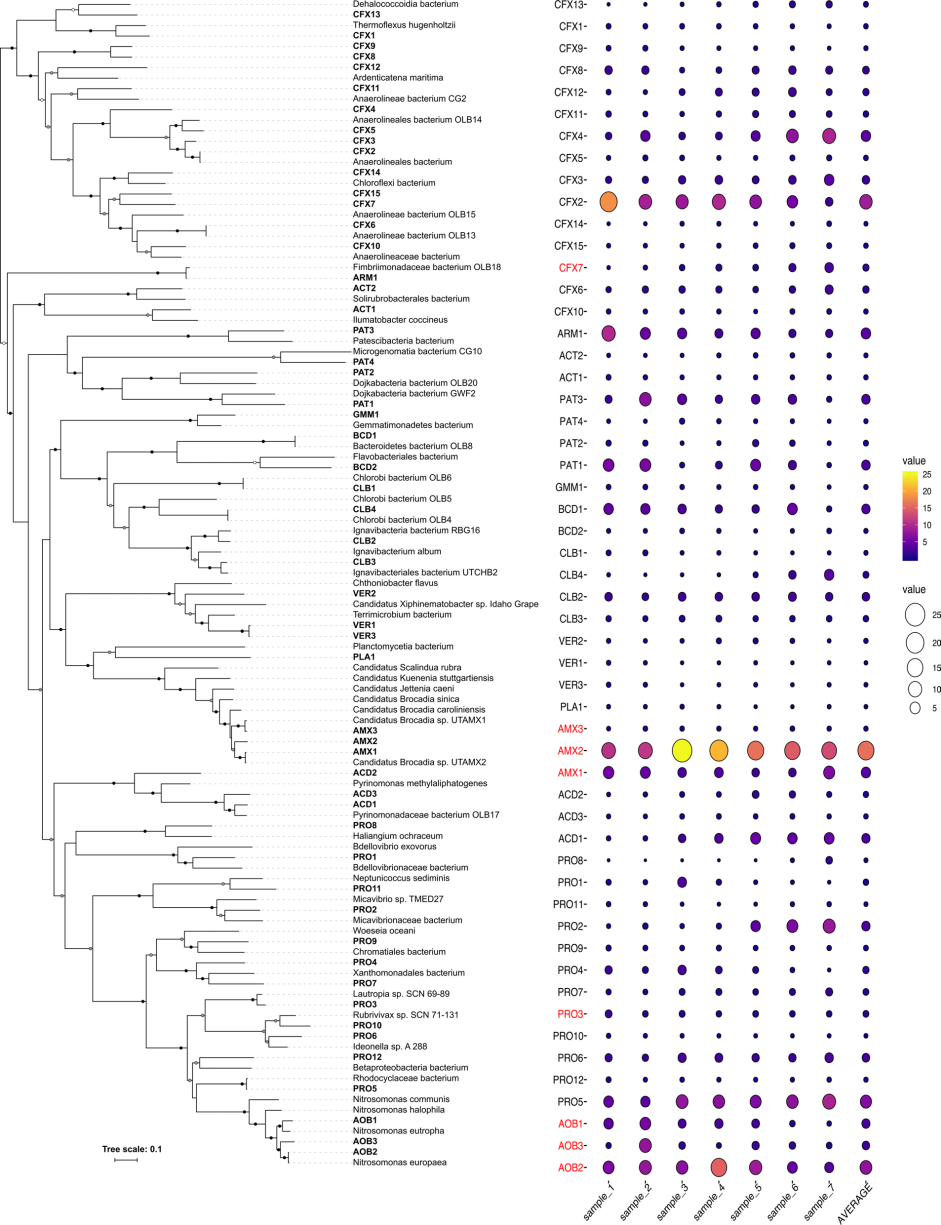
Overview of the recovered MAGs, their relative abundance, dynamics and phylogenetic placement

Whole-community DNA sequencing from seven samples, taken at monthly intervals, from the PNA reactor, generated an average of 2.7 ± 0.5 Gbp high-quality, paired-end sequence data per sample. A total of 55 metagenome-assembled genomes (MAGs) (average completeness and contamination of 81.5% and 1.7%, respectively (Additional file 2: Table S1)) were retrieved from the co-assembled contigs. On average 79% (± 1) of the community metagenome could be assigned to the recovered MAGs indicating a solid coverage of the community by the retrieved MAGs. Temporal dynamics of the community, as inferred from relative MAG abundance, was limited, indicating resilience to the changing aeration conditions (Fig. 1).

### Overall community composition

MAGs were classified as autotrophs if they contained the key genes of any of the previously described carbon fixation pathways (the Calvin-Benson-Bassham (CBB) cycle, the 3-hydroxypropionate cycle, the 3-hydroxypropionate-4-hydroxybutyrate cycle, the reductive tricarboxylic acid (rTCA) cycle or the Wood-Ljungdahl pathway). The community was dominated by non-autotrophic (46 MAGs, ca. 57% abundance) over autotrophic MAG types (9 MAGs, ca. 43% abundance) even though no organic carbon was fed to the system (Additional file 3:Table S2; Fig. 1). The autotrophic MAGs comprised three MAGs each in the AOB (reductive pentose phosphate (CBB)) and AnAOB (Wood-Ljungdahl (WL)) guilds, respectively, and three additional MAGs PRO3, PRO5 and CFX7 carry the CBB and WL pathways, respectively.

### The ammonia oxidizing guilds

Of the core functional guilds, the AOB guild comprises three different MAGs; while AOB2 had an AAI of 97% with *Nitrosomonas europaea*, AOB1 and AOB3 are sufficiently divergent from known genomes to be separate species with *Nitrosomonas eutropha* and *Nitrosomonas europaea* as closest relatives (Additional file 1: Fig. S2). Although differential binning was unable to assign the *amo* and *hao* operons to the appropriate MAGs (see explanation in Additional file 1: Fig. S3), all AOB MAGs have the genes for the ammonia monooxygenase complex (*amoCABDE*) and hydroxylamine oxidase (*hao*), and typical Copper resistance/homeostasis (*copCD*) genes. Genes for NOx reduction were also present: The AOB MAGs contain a Copper-containing nitrite reductase (*nirK*) and cytochrome bc-type complex cNOR (respiratory nitric oxide reductase, *norBC*) (Additional file 3: Table S2).

The AnAOB comprises three different MAGs: AMX1 had an AAI of 99% with *Candidatus* Brocadia fulgida; AMX3 had an AAI of 94% with *Candidatus* Brocadia sp. UTAMX1; AMX2 constitutes a new species, with *Candidatus* Brocadia sp. UTAMX2 as closest relative (Additional file 1: Fig. S2). UTAMX1 and UTAMX 2 were reported as dominant AnAOB in a similar study [19].

All AnAOB MAGs harbor the hydrazine dehydrogenase (*hdh*), hydrazine synthase (*hzsABC*) and nitrate oxidoreductase (*nxrAB*) genes (Additional file 3: Table S2). A gene encoding nitrite reductase (*nirK*), the typical enzyme converting NO_2_^−^ to NO was present in AMX1, but absent from both AMX2 and AMX3 (Additional file 3: Table S2). Besides these genes, multiple copies of *hao*-like genes were present in all AMX MAGs: ten in AMX1 and AMX2 and six in AMX3 (Additional file 1: Fig. S4). Phylogenetic analysis indicates at least eight *hao* clusters congruent with published *hao*. Both AMX1 and AMX2 harbor three homologous copies of a *hao-*like gene previously associated with hydroxylamine oxidation to nitric oxide [19], while AMX3 contains one copy. On the other hand, each of the AMX MAGs have three homologous copies of a *hao*-like gene hypothesized to be involved in nitrite reduction to either nitric oxide or hydroxylamine [19] (Additional file 1: Fig. S4). No *amoA*-like sequences were found that could be assigned to non-autotrophic MAGs; however some *haoA*-like sequences were assigned to non-AOB MAGs (PRO3, PRO5, PRO11); PRO3 has two *haoA* gene paralogues – and both have as closest relative a gene found in *Lautropia* SCN 69-89, previously identified as abundant in nitration/anammox communities[20] and suggested to be a nitrifier-denitrifier.

About half of the recovered MAGs (28/55) harbor genes homologous to *nxr*/*nar* (Additional file 3: Table S2) comprising both the cytoplasmic and periplasmic NxrA/NarG encoding operons (Additional file 1: Fig S5). All of those genes were phylogenetically distinct from those in previously characterized nitrite oxidizing bacteria, however, seven MAGs (PRO3, PRO6, PRO11, PRO12, CFX 1, CFX9, ARM1) encode a periplasmic Nxr that belong to the NOB and AnAOB phyletic group (Additional file 1: Fig. S5). Whether these *nxr*-like genes encode for proteins involved in nitrite oxidation or nitrate reduction remains unknown. Except for PRO3, no MAG contains both a *nxr* operon and a carbon fixation pathway, suggesting the absence of canonical NOBs, but possibility for non-autotrophic nitrite oxidation as noted for an ARM genome retrieved from a anammox microbiome [21].

Even though the recovered MAGs represented approximately 80% of the whole metagenome, we also screened the metagenomic reads to detect possible canonical *nxr* missed during the assembly or binning process. Some reads mapped against canonical *nxr*, especially from *Nitrobacter* spp., although the number was much lower compared to the reads mapping to AnAOB *nxr* (3.0 ± 0.8 reads per million (RPM) vs. 100.2 ± 24.6 RPM). An even lower number of reads mapped to *nxr* from other known NOB (*Nitrolancea* spp.: 0.7 ± 0.2 RPM; *Nitrospira* spp.: 0.3 ± 0.2 RPM; and 0 RPM to *Candidatus* Nitrotoga spp. and *Nitrospinae* spp.) (Additional file 1: Table S3).

### The heterotrophic guilds–NO_x_ respiration

**Fig. 2:**
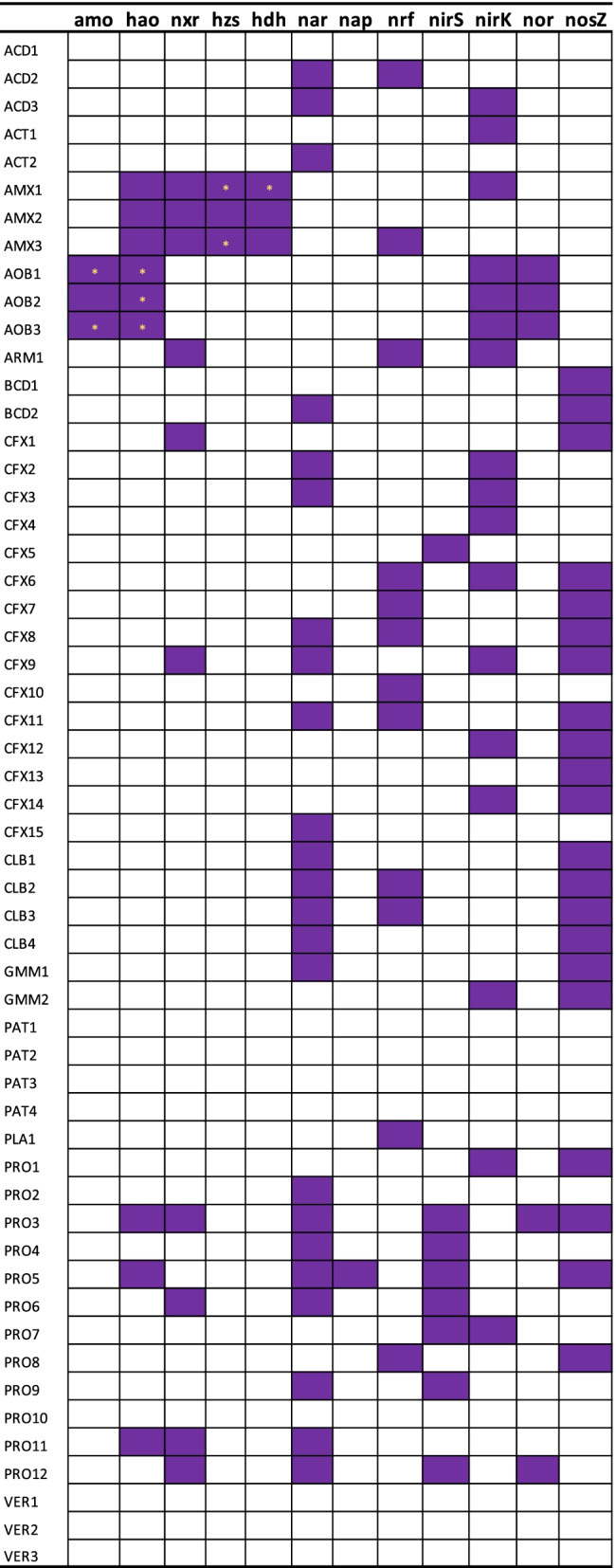
Distribution of nitrogen cycling genes across the MAGs

As more than half of the metagenome was heterotrophic (Fig. 1, Additional file 3: Table S2) it was examined in further detail, especially for its respiratory abilities towards nitrogen oxides (Fig. 2).

Almost half of the MAGs (23/55) encode genes for respiratory nitrate reductase (*narGHIJ*, 28/55), one encodes the periplasmic nitrate reductase gene (*napAB*), some carry the genes for dissimilatory nitrite reduction to ammonia (DNRA) via the periplasmic nitrite reductase (*nrfHA*; 12/55) or the cytoplasmic nitrite reductase (*nirBD*; 2/55) (Additional file 3: Table S2), although complete DNRA (carriage of nitrate reductases gene in addition to *nrfHA* or *nirBD*)) was found in just 5 MAGs (ACD2, CFX8, CFX11, CLB2 and CLB3) (Additional file 3: Table S2). If the above identified *nxr*-like genes (in ARM1 and CFX1) encode a nitrate-reductase, the number of MAGs that can reduce nitrate increases to 25/55.

Of the MAGs with capacity for nitrate reduction, there is only *one* MAG that encodes the complete set of genes for respiration of all reduced nitrogen oxides (the proteobacterial MAG PRO3, which carries, in addition to *narGHI*, also *nirS*, *norBC*, and *nosZ* of the clade I type). Two of the *narGH* encoding MAGs (ACT2 at 0.23% and CFX15 at 0.34% relative metagenome abundance) have no additional genes related to NOx respiration.

The two most abundant heterotrophic MAG, CFX2 (at 8.75% relative abundance) and CLB1 (at 5.20% relative abundance), have, in addition, to a *narGH* operon, solely a *nirK* and a *nosZ* (class II) gene, respectively, clearly indicating incomplete denitrification pathways.

While 23 MAGs encoded genes for respiratory nitrate reduction, 27 MAGs encoded genes for respiratory nitrite reduction (8 *nirS*, 19 *nirK*). Only 7 of these MAGs carried both *nir* and *nar* genes. 8 MAGs encoded genes for nitric oxide reduction (3 with *nor*Z, 5 via *nor*BC). Most of these also carried *nir* genes (7/8), but only half (4/8) carried both *nir* and *nar* genes. Only one MAG carried a class I *nos*Z gene (PRO 3). As stated above, PRO3 was the only MAG with all genes for a complete denitrification pathway. On the other hand, many MAGs encoded a class II *nosZ* gene (20/55), revealing a very high genomic potential for high-affinity N_2_O reduction. None of these MAGs carried genes for NO reduction (i.e. the *norBC* or *norZ* genes), while 7 of these MAGs carried genes for nitrite reduction (*nirS* or *nirK*, CFX6, PRO1, BCT2, PRO5, CFX9, BCT5, BCT6) or for nitrate reduction (*narGHI*, IGN1, IGN2, PRO5, BCG1, CFX8, BCT11, BCD2), respectively.

**Fig. 3:**
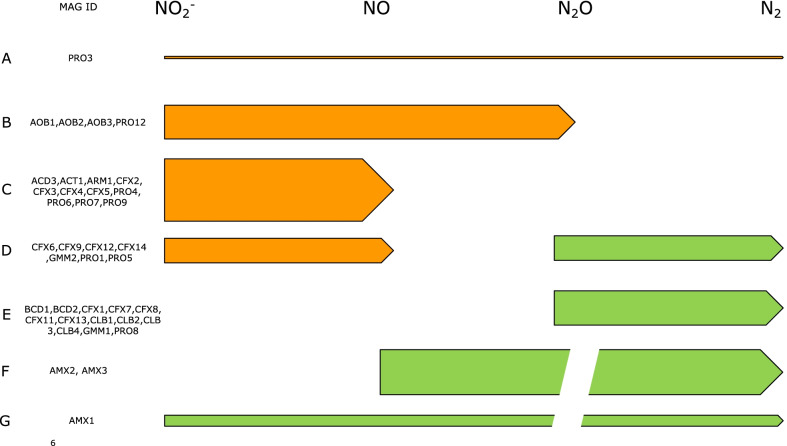
Overview of the genomic potential for production and consumption of NOx intermediates as distributed across the MAGs

While MAGs with the capability of NO_3_^−^ to NO_2_^−^ reduction and N_2_O to N_2_ were abundant, MAGs with incomplete denitrification pathways prevailed (Figs. 2 and 3). MAGs that were characterized as NO_2_^−^ to N_2_O reducers consisted of AOB, consistent with process observations [22]. In addition, a large number of MAGs with potential to exclusively reduce NO_2_^−^ to NO or with potential for NO_2_^−^ to NO, and N_2_O to N_2_ reduction were recovered. Several MAGs with potential to consume NO were retrieved, but especially the AnAOB MAGs AMX2 and AMX3 stand out as they did not encode the expected nitrite reductase (*nirK*). The abundance of MAGs with genetic potential for NO production (and not consumption) suggest that NO is exchanged in the community; AMX2 and AMX3 are the obvious NO consumers, suggesting growth of AnAOB on NO (not NO_2_^−^) as electron acceptor as recently documented [23]. While there are MAGs with the exclusive ability to reduce N_2_O, most of them also encode genes (*nar* or *nrf*) allowing for NO_3_^−^ to NO_2_^−^ or NO_2_^−^ to NH_4_^+^ reduction (Additional file 3: Table S2).

### Auxotrophy across MAGs

**Fig. 4:**
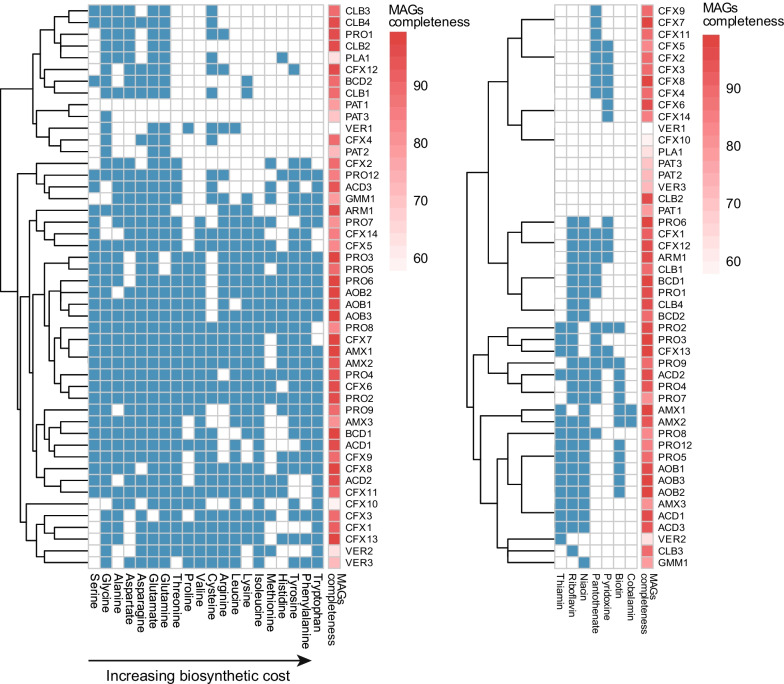
Presence/absence of biosynthetic pathways for amino acid and B-vitamins across MAGs

The heterotrophs in anammox or nitritation-anammox systems can either provide growth factors to [14] or depend on growth factors from [15] the autotrophic community members. Hence, we examined the genomic potential for synthesis of AA and B-vitamins across the MAGs. Of all the recovered MAGs, only one (PRO4) was fully prototrophic for its AA; all other MAGs were at least auxotrophic in one and up to all (PAT1) amino acids (Fig. 4). The dominant autotrophs (AOB) were nearly prototrophic, all three missing the cysteine, plus the alanine (AOB2) and leucine (AOB1) biosynthesis pathway, respectively. Similarly, among the AnAOB, AMX1 and AMX2 only missed the methionine biosynthetic pathway, while AMX3, in addition, lacked complete histidine and proline biosynthetic pathways. Similarly, none of the MAGs was totally prototrophic for its B-vitamin synthesis, with some MAGs (PAT1, PAT3, PRO10, PLA1, VER1, VER2, VER3 GMM2) completely devoid of this genetic potential (Fig. 4). The potential for cobalamin biosynthesis (Vit B12) was only retrieved in the AnAOB MAGs AMX1 and AMX2. Correlations between the degree of prototrophy and MAG completeness or MAG abundance were low: both nearly complete MAGs (> 95%) and very rare MAGs (< 5%) ranged in AA prototrophy from 25 to 100%; yet the most abundant MAG (AMX2) was the most prototrophic (Additional file 1: Fig. S6).

## Discussion

### Performance and NOB suppression

PNA has been successfully attained using various reactor configurations/operations and resulting biomass morphologies [5]. In general, attached growth (biofilms, aggregates) seems necessary to obtain sufficient AnAOB in the system, yet the same systems are challenged by the retention of unwanted NOB. NOB control is easier to obtain in suspended growth (or hybrid suspended/attached growth) systems [11]. Here we document that granular systems—obtained by sequential feeding and subjected to intermittent aeration—permit both retention of AOB and AnAOB (regulated by diffusional transport of O_2_, NH_4_^+^ and NO_2_^−^[24])) but also control NOB density. While causes for repression of NOB by aeration switching have been proposed [12, 25–27], direct proof has remained elusive and this operational control towards microbiome engineering remains heuristic.

### Presence and diversity of AOB and AnAOB

In the resulting PNA community, operated on a synthetic feed with NH_4_^+^ as the sole energy source, AOB and AnAOB amounted to 13% (stdev: 5%) and 22% (stdev: 6%) of the community (as fractions of the metagenome). This is similar to the fractions observed in other PNA communities by Speth et al. (AOB max 4% AnAOB max 20%) and Wang et al. (AOB ca. 25% AnAOB ca. 40%) [28]. While Speth et al. [29] and Wang et al. [28] identified only one MAG as AOB and AnAOB, we identified 3 MAGs each that could be classified as AOB and AnAOB. This may, in part, be due to the higher fraction of the MG that could be assigned to the different MAGs (79% here vs only 59% in [29]); even though the distribution across MAGs was more equitable in our study compared to Wang et al. [28]. The dominant AnAOB and AOB in the current system were closely related to *Candidatus* Brocadia sp., and *Nitrosomonas europaea* and *N. eutropha* as typically found in these highly loaded synthetic PNA or (for AnAOB) anammox communities [14, 19, 28, 29].

### Absence of NOB

Although 16S rRNA gene targeted qPCR ([16, 30]) and 16S rRNA gene amplicon targeted community analysis (Additional file 1: Fig. S1) indicated a small *Nitrospira* presence (< 0.5%), no MAGs encoding autotrophic nitrite oxidation were recovered, and the presence in the whole metagenome was also minimal (ca. 4 RPM mapped to canonical NOB *nxr*). Speth et al. [29], on the other hand, detected a *Nitrospira* MAG (at 1.6 to 2.8%); yet also Wang et al. did not detect NOB MAGs in their PNA community MG [28]. As both our study and [28] applied sequential (instead of continuous low-rate) aeration to support the PNA community, this may be an effective strategy for NOB counter selection. We also note that, in fact, *Nitrospira* detection based on 16S rRNA gene or on *nxrA*, as done here, is not necessarily indicative of the presence of strict NOB since comammox *Nitrospira* have also been recovered from PNA systems [31]. Therefore the low fraction of *Nitrospira* we detect might be an overestimate of NOB abundance.

### The types of HB and the role of auxotrophy vs. prototrophy of HB

Heterotrophic bacteria were abundant in this study (57% of MG vs autotrophs 43%); consistent with other studies [14, 15, 29]. They were distributed across a diverse set of phylotypes but with notable abundance in Chloroflexi (MAG CFX 1-15 at 23%), Ignavibacteriales/Chlorobi (MAG CLB 1-4 at 5%), Armatimonadates (MAG ARM1 at 4%), Bacteroidetes/Flavobacteria (MAG BCD 1,2 at 4%), and a few Proteobacteria (MAG PRO 2 and 6 at 3 and 2%). These taxa are all typically found in PNA and/or anammox communities. Heterotrophs are assumed to be supported by soluble microbial products actively or passively released by autotrophic PNA members [32]. In addition, others have suggested heterotrophs as essential in providing growth factors to autotrophs [14, 33], a claim not consistent with our findings. Auxotrophy for AA and vitamin biosynthesis were present in both autotrophs as heterotrophs; the most auxotrophic MAGs were heterotrophs, and AnAOB MAGs were the only MAGs encoding potential for cobalamin biosynthesis (Fig. 4). Clearly mutual dependencies beyond exchange of N species drive the composition of the PNA microbiome [15, 34].

### Denitrification pathways

The current metagenome analysis indicates that heterotrophic MAGs have varying abilities for NOx respiration, with only one MAG encoding a complete denitrification pathway. Similar observations were made before: rare MAGs encoding full denitrification, but wide potential (and expression) for NO_3_^−^ to NO_2_^−^ respiration across multiple MAGs [15, 19, 29]. These observations have supported the notion that heterotrophs in PNA systems support a nitrite loop [35]. Our analysis results suggest, in addition, an abundance of MAGs with NO as the predicted end product of NO_x_ respiration. In combination with the fact that the two AnAOB MAGs AMX2 and AMX3 (Additional file 3: Table S2) lack NIR encoding genes, this raises the possibility for NO cycling between autotrophic and heterotrophic MAGs. While the ability to support anaerobic ammonium oxidation supported by NO reduction (instead of NO_2_^−^) has been shown in pure culture [23], direct proof in a PNA microbiome awaits confirmation.

In conclusion, our metagenomics analysis indicates that intermittent aeration is a highly-efficient control strategy to suppress NOB presence in a PNA process. The resulting microbiome presents mutual dependencies between the AOB and AnAOB autotrophs and heterotrophs, and a N cycle network that involves NO exchange.

## Materials and methods

### Sample collection and extraction of DNA

Mixed biomass was collected as 2 mL grab samples at the end of each set frequency period (monthly) and at the end of a react phase. The samples were centrifuged at 10,000xg, supernatant removed and stored as pellets at − 20 °C until DNA extraction. DNA was extracted using the MP FastDNA Spin Kit (MP Biomedicals LLC, Solon, USA) following manufacturer instructions. DNA concentration and quality were measured by NanoDrop (NanoDrop Technologies, Wilmington, USA).

### Library preparation, sequencing and de novo assembly

DNA-shearing and library preparation were conducted based on the NEXTflex Rapid DNA-Seq Kit, V13.08 (Bioo Scientific, Austin, TX, USA) as described [36]. Sequencing was performed as a 100-bp pair-end run on HiSeq 2000 (Illumina Int., San Diego, CA, USA) at BGI (Copenhagen, Denmark). *FastQC* [37] was used for quality control, while *Trimmomatic* v0.22 [38] was run to remove adapters and trim the reads (threshold quality = 30; minimum length = 45). Assembly of high-quality reads from each sample into contigs was performed using *IDBA-UD* [39] with default parameters.

### Metagenomic assembled genomes recovery and annotation

Genome binning was conducted based on pentanucleotide signatures using VizBin [40] and differential coverage using mmgenome [41]. Resulting metagenomic assembled genomes (MAGs) were manually evaluated through contig depth and GC content. Completeness and potential contamination of each MAG was evaluated using CheckM [42]. When the same draft MAG was obtained from several samples, comparison between them was implemented, retaining the one best assembled, most complete, and with the lowest contamination. MAGs with at least 70% of completeness, or an inferior number without contamination were further analyzed. The relative abundance of the MAGs was calculated with CoverM v0.6.1 (https://github.com/wwood/CoverM).

### Taxonomic and functional annotation

MAGs were classified using the classify workflow of the GTDB-Tk v.0.1.3 tool [50]. Predicted coding sequences retrieved using *Prodigal* 2.50 [43] were annotated using USEARCH [44] *-ublast* against the manually created databases of reference proteins encoded by genes of interest (best hit with E < 1e^−5^, Bitscore > 60 and sequence similarity > 30%). Furthermore, to confirm protein functional assignment, Kyoto encyclopedia of genes and genomes (KEGG) annotations of the predicted proteins in each CG were obtained using the WebMGA server [45]. Presence of the complete operon was evaluated in MG-RAST [46]. The presence of carbon fixation pathways in each MAG was evaluated using METABOLIC [47]. In addition, GhostKOALA was used to assign KEGG orthology (KO) to each predicted ORFs of retrieved MAGs [48]. KEGG mapper was then used to process KO annotation results and reconstruct metabolic pathways of the retrieved MAGs. The presence/absence of an amino acid or vitamin biosynthesis pathway was estimated based on the encoded genes for the steps within a given pathway. If a reaction step could be catalyzed by more than one enzyme, the presence of a gene encoding one of the enzymes was regarded as the presence of this reaction step. The presence of a metabolic pathway in a given MAG was estimated as follows: If a pathway includes less than two steps, all steps are required to be encoded. If a pathway included more than two steps, only one missing step was allowed.

To detect canonical *nxr* in the whole metagenome, VSEARCH (–usearch_global, –id 0.85, –query_cov 0.9) [49] was used to map filtered metagenomic reads against a custom database containing the *nxrA* gene of previously described canonical NOB (*Candidatus* Nitrospira defluvii, *Nitrospira lenta*, *Nitrobacter hamburgensis*, *Nitrobacter winogradskyi*, *Nitrolancea hollandica*, *Candidatus* Nitrotoga sp. KNB and *Candidatus* Nitronauta litoralis). Same analysis was conducted to detect *nxr* from AnAOB (using the *nxrA* from *Candidatus* Brocadia pituitae and *Candidatus* Kuenenia stuttgartiensis).

### Phylogenetic analysis

Phylogenetic analyses of the recovered MAGs were conducted with the GTDB-Tk v.0.1.3 tool [50] using the de novo workflow with a set of 120 single copy marker proteins and the genome taxonomy database (GTDB) [51]. Predicted NxrA/NarG and HaoA/HzoA amino-acid sequences were independently aligned with reference sequences using MUSCLE [52]. These alignments were used to construct maximum likelihood trees using RAxML v. 8.2.11 (the number of bootstraps was determined using the autoMRE option) [53]. For NxrA/NarG, the tree was built using the PROTGAMMAILGF model of sequence evolution, while for HaoA/HzoA the tree was constructed using the PROTGAMMAIWAG model. In both cases the best model was determined using ProtTest v. 3.4.2 [54]. All trees were visualized using the online web tool from the Interactive Tree of Life (iTol) [55].

## Supplementary Information

**Additional file 1:** Supplemental Information, Supplementary Figures and Table S3.

**Additional file 2: Table S1:**  Quality,  abundance and taxonomy of the recovered metagenome-assembled genomes.

**Additional file 3: Table S2:** Overview of the genetic content of the recovered metagenome-assembled genomes.

## Data Availability

16S rRNA gene sequences, shotgun metagenomic sequences and metagenome-assembled genomes retrieved from the bioreactor have been deposited at NCBI under the project PRJNA791618.
