# Supplementary material for: Efficient management of the nitritation-anammox microbiome through intermittent aeration: absence of the NOB guild and expansion and diversity of the NOx reducing guild suggests a highly reticulated nitrogen cycle
Source: Environ Microbiome. 2022 Jul 22;17:39. doi: 10.1186/s40793-022-00432-2 (PMC9306079; doi:10.1186/s40793-022-00432-2)
Supplement: Supplementary file 1 — Additional file 1: Supplemental Information, Supplementary Figures and Table S3. [file 40793_2022_432_MOESM1_ESM.pdf]

Supplemental Information

Title

Efficient management of the nitritation-anammox microbiome through intermittent aeration: Loss of the NOB guild and expansion and diversity of the NOx reducing guild suggests a highly reticulated nitrogen cycle.

Authors

Alejandro Palomo[1], Bruna Daniela Azevedo da Silva[1], María Touceda Suárez[1], Carlos Domingo-Félez[1], A. Gizem Mutlu[1], Arnaud Dechesne[1], Yulin Wang[2], Tong Zhang[2], Barth F. Smets[1]

[1] Microbial Ecology and Technology Lab, Department of Environmental Engineering, Technical University of Denmark, Kgs Lyngby, DK

[2] Environmental Microbiome Engineering and Biotechnology Laboratory, Department of Civil Engineering, The University of Hong Kong, Hong Kong SAR, People's Republic of China

Reactor operation/performance summary

The PNA reactor was a sequencing batch reactor with 8-hour cycles consisting of anoxic feeding, reaction with continuous mechanical stirring and intermittent aeration, settling, discharge, and idle phases, with a hydraulic retention time of 16 hours. The seeding biomass originated from a full-scale moving bed biofilm reactor treating anaerobic digester supernatant via PNA (ANITA Mox). The reactor was fed an anoxic synthetic medium modified after [1] with deionised water and NH_4HCO_3 as the sole energy source, at a volumetric loading of 750 mg N/L·day. The solids retention time was set to 100 days by daily manual wasting of mixed reactor biomass. The oxygen to ammonium loading was nominally 1.09 g O_2/g N, increased to 1.20 g O_2/g N during the final high frequency period to regain TN-removal efficiency while dissolved oxygen concentration was below detection limit throughout. The reference aeration frequency of 3, length of aerated/non-aerated period and the air flow rate were attained during 19-month operation following an aeration regimen based start-up protocol. The reactor was operated at 30°C. Nitrogen species NH_4^+, NO_2^-, NO_3^- were monitored for all the cycles and throughout one cycle at the end of each frequency setting period to determine the performance per cycle and during redox cycling. In-cycle liquid NO and N_2O levels were also monitored per frequency setting period.

Nitrite accumulation and N_2O production were observed during aerated periods, overall both decreased with increasing frequency. Average aggregate size increased and settling properties ($SVI_{5/30}$) improved with increasing aeration frequency. Based on 16S rRNA gene targeted qPCR and FISH, the community architecture switched from size-segregated AOB (<90μm) and AnAOB (>90μm) with bi-modal aggregate size distribution to uni-modal stratified AOB-AnAOB coaggregates. Other (non-autotrophic) guilds had initially higher abundance in <90μm size fraction, with increasing aeration frequency their relative abundance increased in all size fractions.

Table S1. Quality, abundance, and taxonomy of the recovered metagenome assembled genomes (attached xl spreadsheet)

Table S2 Overview of the genetic content of the recovered genomes (attached xl spreadsheet)

Table S3. Reads per million (RPM) mapped against AnAOB and canonical *nxrA*.

	t0	t1	t2	t3	t4	t5	t6	Mean	StDv
AnAOB	78.3	89.3	144.1	123.7	97.0	79.9	89.2	100.2	24.6
Nitrobacter	4.4	3.4	2.3	2.9	3.3	2.4	2.3	3.0	0.8
Nitrolancea	1.1	0.6	0.5	0.5	0.8	0.5	0.7	0.7	0.2
Nitrospira	0.1	0.2	0.2	0.1	0.6	0.5	0.3	0.3	0.2
Nitrotoga	0.0	0.0	0.0	0.0	0.0	0.0	0.0	0.0	0.0
Nitronauta	0.0	0.0	0.0	0.0	0.0	0.0	0.0	0.0	0.0

PCR for pyrosequencing

Ten nanograms of extracted DNA was PCR amplified using Phusion (Pfu) DNA polymerase (Finnzymes, Finland) and 16S rRNA gene-targeted universal primers PRK341F (5'-CCTAYGGGRBGCASCAG-3') and PRK806R (5'- GGACTACNNGGGTATCTAAT-3') (Yu et al. 2005). PCR was performed as described elsewhere [2]. The adapter- and sample-specific tag addition and pyrosequencing were done as described previously [3].

Bioinformatic analysis

All raw 16S rRNA sequence were processed in in DADA2 v1.2 [4] following the SOP for single ended 454 reads. We obtained an average of ~7000 denoised ASVs per sample. These were assigned a putative taxonomic affiliation using IdTaxa as implemented in the package DECIPHER [5] using MIDAS4.8.1 database as reference [6]. Graphical visualisation of taxonomic composition was done with "phyloseq" package in R environment [7].

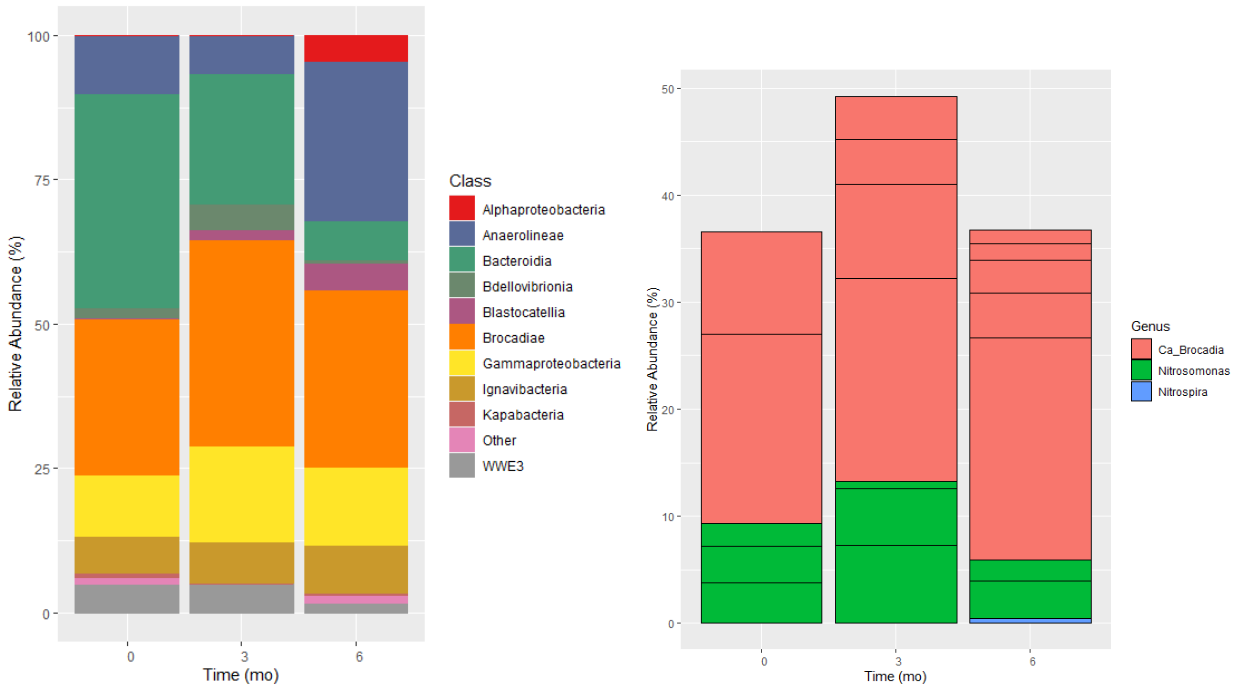

Figure S1 16S rRNA amplicon-inferred community composition at time 0, 3, 6 months for total community (left) and the AOB and NOB-oxidizing guilds (right). The 'Other' category comprises all ASVs assigned to the classes contributing less than 2% of the total.

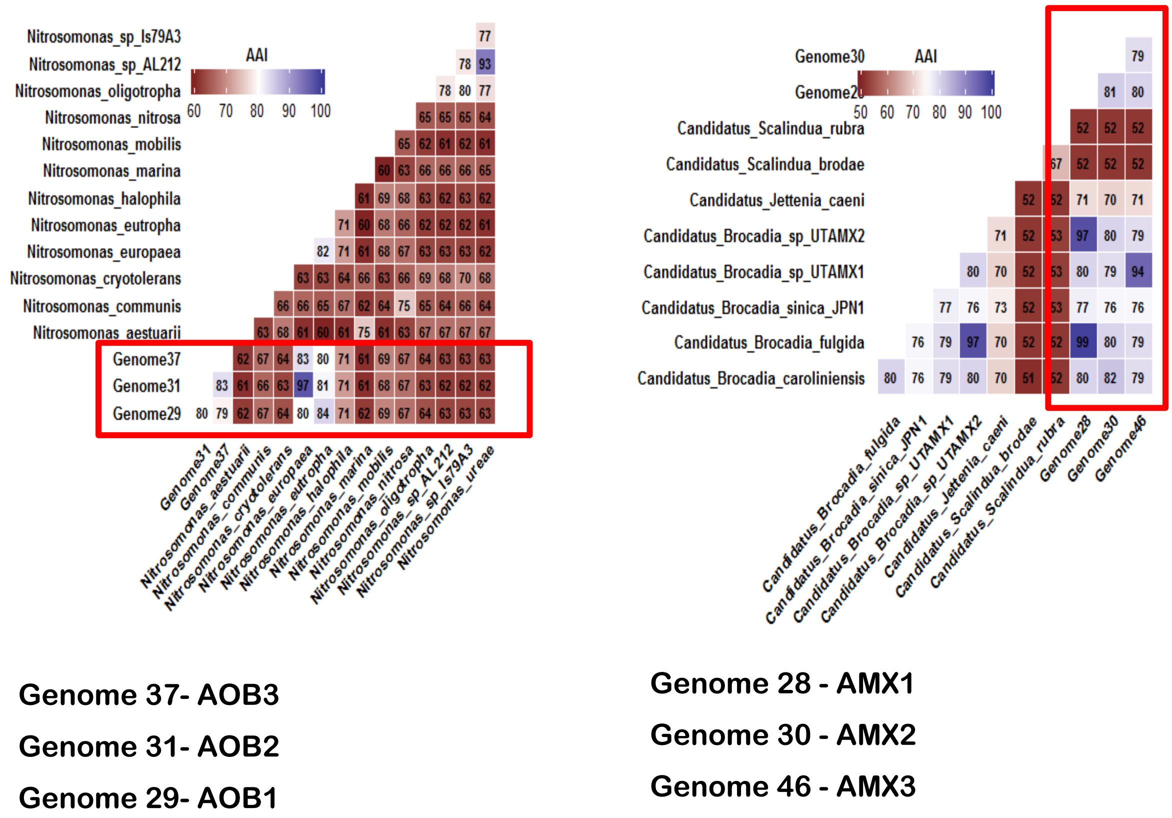

Figure S2 Pairwise AAI comparison of retrieved AeAOB and AnAOB MAGs against reference species.

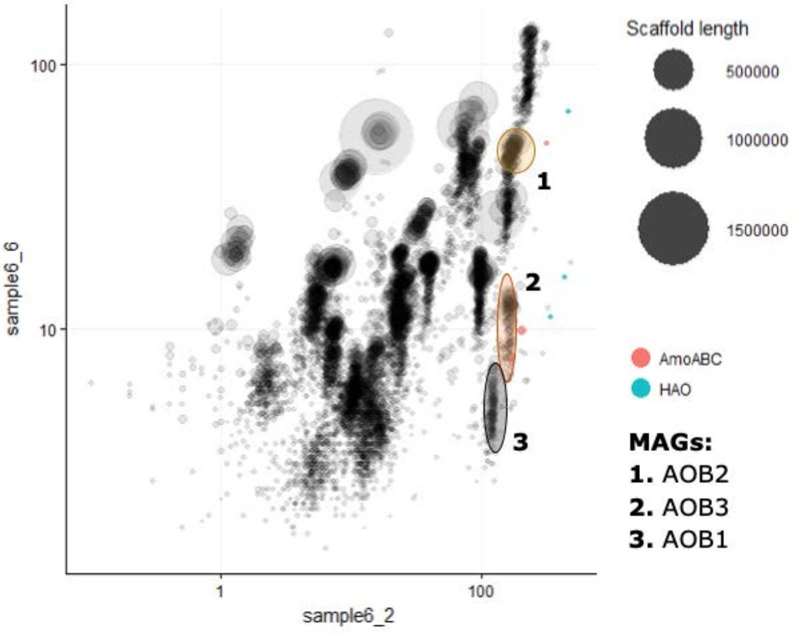

Figure S3 Differential coverage plot of sample6 using two different subsamples of the total reads. *amo* and *hao* genes are highlighted and the *Nitrosomonas* genomes position identified.

The AMO operon was solely detected in AOB3 MAG, and not in AOB1 and AOB2. Similarly, no AOB MAG contained the HAO operon. However, three AMO and HAO operons where found when searching the whole metagenome, instead of only the recovered MAGs. A likely explanation is that the typical presence of 2-3 almost identical copies of the *amo* and *hao* genes in *Nitrosomonas* spp. [8] can result in a singular unique copy during *de novo* assembly. This results in artificially increased sequencing depth of the mentioned unique assembled operons. As this genomic region then has a higher depth than the rest of the genome proper binning is not possible when differential coverage binning methods are used (as in this study) [9]. In fact, when the AMO and HAO operons were highlighted in a differential coverage plot, both genomic regions were closely placed to the AOB bins (Fig. S3). Therefore, we concluded that the missing operons in the AOB MAGs are artefacts and that each MAG harbours the AMO and HAO operons.

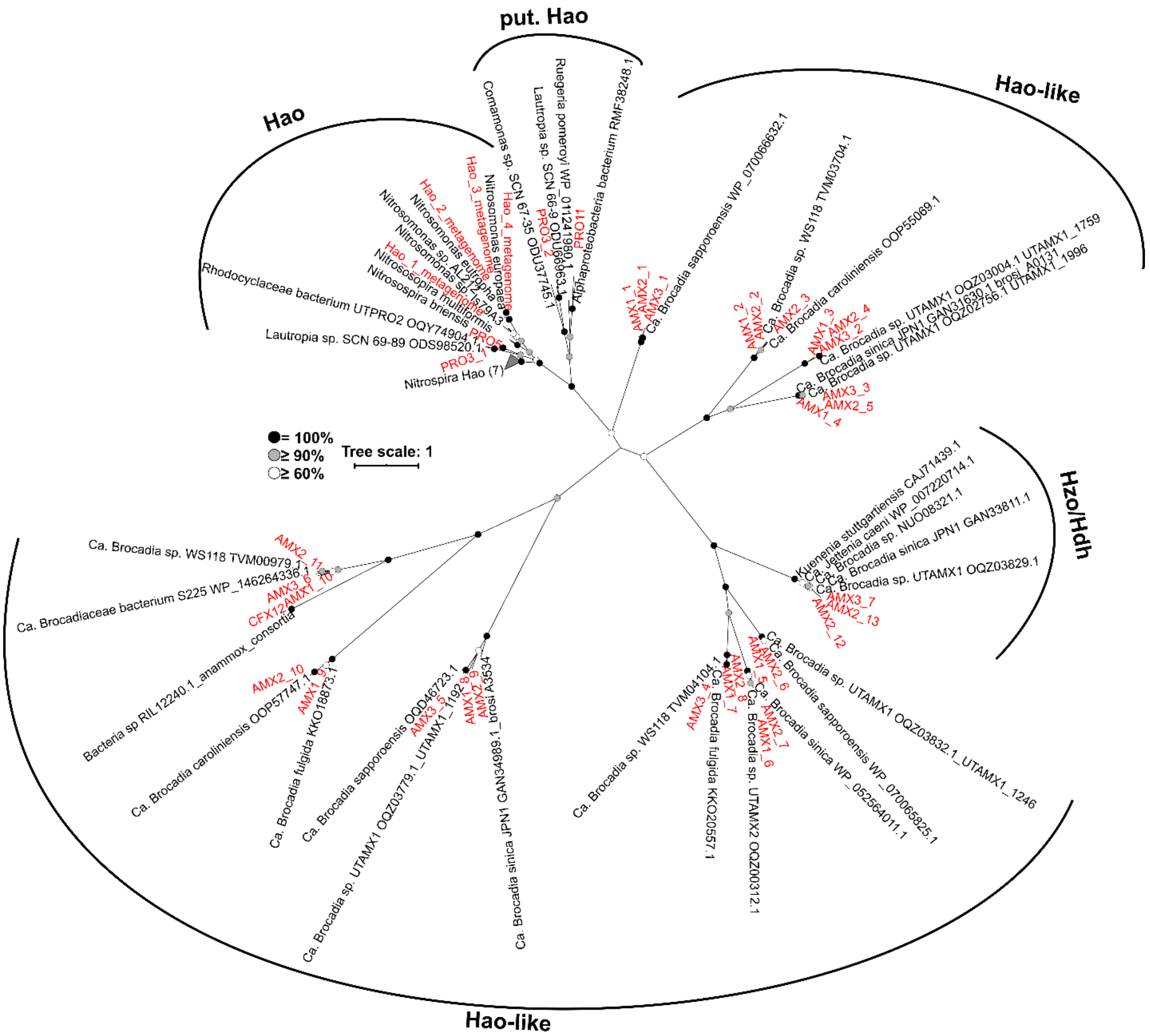

Figure S4 Phylogeny of the retrieved *hao* and *hao*-like genes

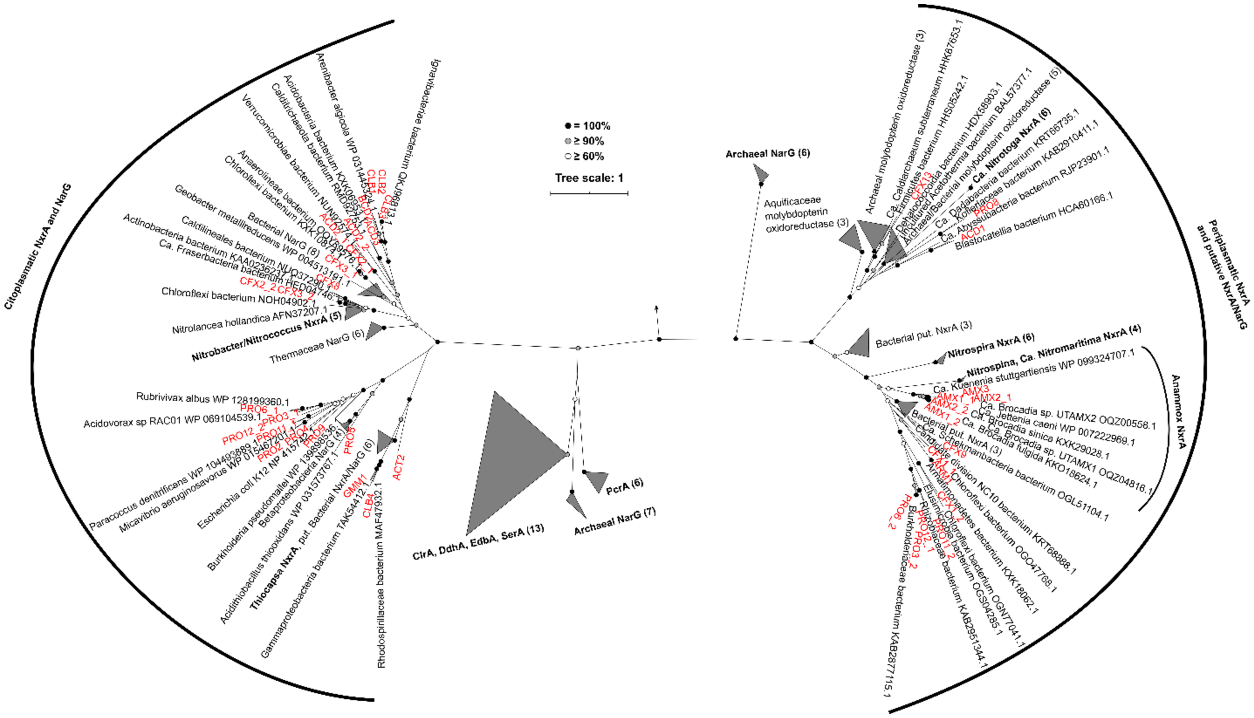

Figure S5 Phylogeny of the retrieved *nxr* and *nxr*-like genes

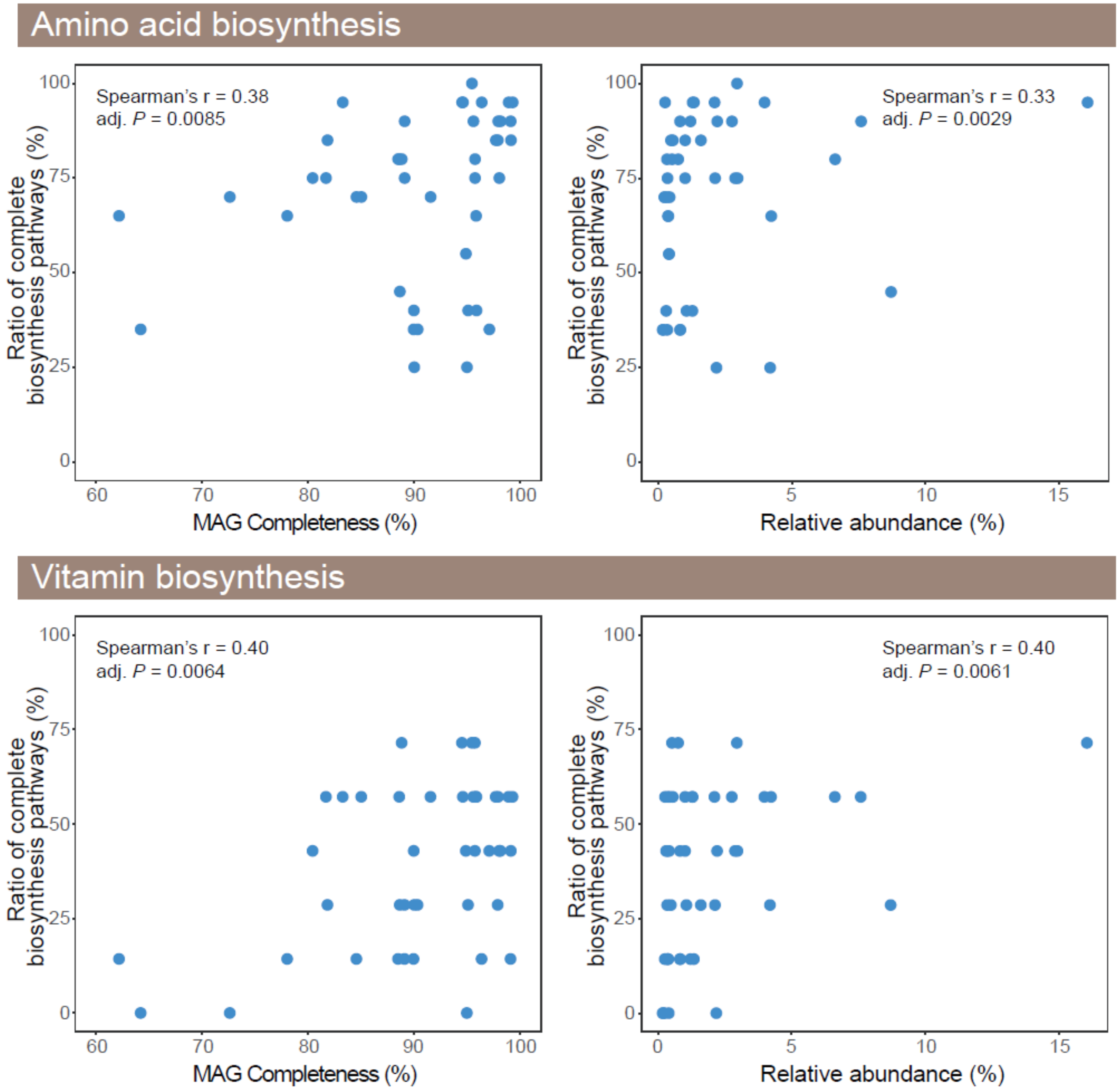

Figure S6. Relation between completeness of biosynthetic pathways for amino acid and B-vitamins across MAGs, MAG completeness, and MAG abundance.

References

1. van de Graaf AA, Mulder A, de Bruijn P, Jetten MS, Robertson LA, Kuenen JG. Anaerobic oxidation of ammonium is a biologically mediated process. *Appl Env Microbiol* 1995; **61**: 1246–1251.

2. Pellicer-Nàcher C, Sun S-PS, Lackner S, Terada A, Schreiber F, Zhou Q, et al. Sequential Aeration of Membrane-Aerated Biofilm Reactors for High-Rate Autotrophic Nitrogen Removal: Experimental Demonstration. *Environ Sci Technol* 2010; **44**: 7628–7634.

3. Farnelid H, Andersson AF, Bertilsson S, Al-Soud WA, Hansen LH, Sørensen S, et al. Nitrogenase Gene Amplicons from Global Marine Surface Waters Are Dominated by Genes of Non-Cyanobacteria. *PLoS One* 2011; **6**: e19223.

4. Callahan BJ, McMurdie PJ, Rosen MJ, Han AW, Johnson AJA, Holmes SP. DADA2: High-resolution sample inference from Illumina amplicon data. *Nat Methods* 2016; **13**: 581–583.

5. Murali A, Bhargava A, Wright ES. IDTAXA: A novel approach for accurate taxonomic classification of microbiome sequences. *Microbiome* 2018; **6**: 1–14.

6. Dueholm MKD, Nierychlo M, Andersen KS, Rudkjøbing V, Knutsson S, et al. MiDAS 4: A global catalogue of full-length 16S rRNA gene sequences and taxonomy for studies of bacterial communities in wastewater treatment plants. *Nat Commun* 2022; **13**: 1–15.

7. McMurdie PJ, Holmes S. Phyloseq: An R Package for Reproducible Interactive Analysis and Graphics of Microbiome Census Data. *PLoS One* 2013; **8**.

8. Kozlowski JA, Stieglmeier M, Schleper C, Klotz MG, Stein LY. Pathways and key intermediates required for obligate aerobic ammonia-dependent chemolithotrophy in bacteria and Thaumarchaeota. *ISME J* 2016; **10**: 1836–1845.

9. Zhou W, Chen T, Zhao H, Eterovic AK, Meric-Bernstam F, Mills GB, et al. Bias from removing read duplication in ultra-deep sequencing experiments. *Bioinformatics* 2014; **30**: 1073–1080.
